# SYSU-6, A New 2-D Aluminophosphate Zeolite Layer Precursor

**DOI:** 10.3390/molecules24162972

**Published:** 2019-08-16

**Authors:** Jiang-Zhen Qiu, Long-Fei Wang, Jiuxing Jiang

**Affiliations:** MOE Key Laboratory of Bioinorganic and Synthetic Chemistry, School of Chemistry, Sun Yat-sen University, Guangzhou 510275, China

**Keywords:** aluminophosphate, 2-D zeolite, SYSU-6

## Abstract

Two-dimensional aluminophosphate is an important precursor of phosphate-based zeolites; a new Sun Yat-sen University No. 6 (SYSU-6) with |Hada|_2_[Al_2_(HPO_4_)(PO_4_)_2_] has been synthesized in the hydrothermal synthesis with organic structure-directing agent (OSDA) of *N*,*N*,3,5-tetramethyladamantan-1-amine. In this paper, SYSU-6 is characterized by single-crystal/powder X-ray diffraction, scanning electron microscopy, energy-dispersive X-ray analysis, transmission electron microscopy, infrared and UV Raman spectroscopy, solid-state ^27^Al, ^31^P and ^13^C magic angle spinning (MAS) NMR spectra, and elemental analysis. The single-crystal X-ray diffraction structure indicates that SYSU-6 crystallized in the space group *P*2_1_/*n*, with a = 8.4119(3), b = 36.9876(12), c = 12.5674(3), α = 90°, β = 108.6770(10)°, γ = 90°, V = 3704.3(2) Å^3^, Z = 4, R = 5.12%, for 8515 observed data (I > 2σ(*I*)). The structure has a new 4,12-ring layer framework topology linked by alternating AlO_4_ and PO_4_ tetrahedra. The organic molecules reside between the layers and are hydrogen-bonded to the inorganic framework. The new type of layer provides a greater opportunity to construct zeolite with novel topology.

## 1. Introduction

Following the discovery of microporous aluminophosphates (AlPO) [1], there has been considerable interest in developing new aluminophosphate compounds with novel framework structures because of their potential application in catalysis, adsorption, and separation. Among AlPO-based molecular sieves, 2-dimensional (2-D) layered structures are significant since they have more accessible surfaces in the applications [2]. Moreover, they can be used as precursors to synthesize 3-dimensional (3-D) framework, such as zeolite [3,4].

As early as 2003, Yu et al. [5,6] provided an excellent review of the rich structure chemistry of aluminophosphate including 3D neutral open framework, anionic open framework, low-dimensional anionic framework. Furthermore, the same group built a database to enumerate the scientific reports of aluminophosphate with a topological independent structure [7,8]. The structural and compositional richness of AlPOs is attributed to the diverse coordination of Al and P atoms. The majority of AlPO molecular sieves are based on a four-connected network of corner-sharing tetrahedra, i.e., AlO_4b_ and PO_4b_ (b: bridging oxygen between Al and P). Whereas, the larger ionic radii renders five or six coordination of Al (i.e., AlO_3b_O_1t_, AlO_4b_, AlO_5b_, AlO_4b_O_1t_, AlO_4b_O_2t_, AlO_4b_(H_2_O)_2_, AlO_4b_O_1t_(H_2_O), AlO_5b_O_1t_, and AlO_6b_, AlO_3b_F_1t_, AlO_4b_F_1t_, AlO_4b_F_2t_, AlO_5b_F_1t_, AlO_4b_F_1b_, AlO_4b_F_2b_, AlO_4b_F_1b_F_1t_), while the P coordinations are solely four in the forms of PO_4b_, PO_3b_O_t_, PO_2b_O_2t_, PO_b_O_3t_ (b: bridging O between Al and P; t: terminal O). Layered AlPOs exhibit a diversified Al:P ratios (i.e., 1:1, 1:2, 2:3, 3:4, 4:5 and 13:18) as aluminum can adopt a configuration of tetrahedral, bipyramidal or octahedral, etc. [7]. For comparison, here we only address the 2-D AlPO with Al:P ratio 2:3 and framework composition [Al_2_(HPO_4_)*_x_*(PO_4_)_3–*x*_]^(3–*x*)−^(*x* = 1 or 2). Table 1 summarizes 13 examples currently known, including the one described below, which can be assembled from combinations of AlO_4_, AlO_5_, AlO_6_, and PO_4_ polyhedra. 13 distinct layer topologies have been identified, all of which contain 4-membered rings of alternating Al- and P-based polyhedra together with 6-rings, 8-rings, or 10-rings. In the present work, we report the synthesis and characterization of Sun Yat-sen University No. 6 (SYSU-6) with molecule formula |Hada|_2_[Al_2_(HPO_4_)(PO_4_)_2_], a new two-dimensional aluminum phosphate with an Al:P ratio of 2:3, containing interlayer organic structure-directing agent (OSDA), *N*,*N*,3,5-tetramethyladamantan-1-ammonium (abbreviated as ada) cations. In this paper, the relationship between SYSU-6 and 2.2.3.2.002 is discussed, due to the similarity of the structures.

## 2. Results and Discussion

**Figure 1:**
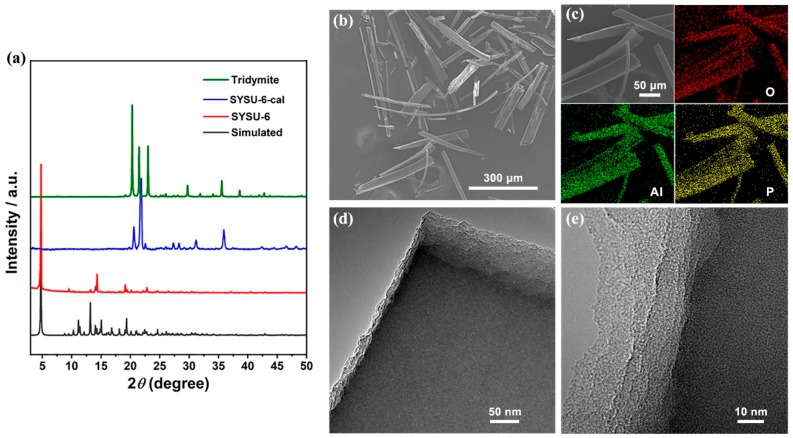
(**a**) Powder X-ray diffraction of Sun Yat-sen University No. 6 (SYSU-6) and SYSU-6-cal; (**b**) scanning electron microscopy (SEM) image of SYSU-6. Indication of the phase purity of SYSU-6; (**c**) SEM and corresponding mapping images for O, P, and Al elements; (**d**,**e**) transmission electron microscopy (TEM) images of SYSU-6.

The experimental powder X-ray diffraction (PXRD) pattern and the simulated X-ray diffraction (XRD) pattern of compound SYSU-6 based on single-crystal X-ray diffraction structure are shown in Figure 1a. The peak positions of both patterns were in agreement with each other, suggesting the phase purity of the as-synthesized compound. Unfortunately, SYSU-6 shows poor thermal stability. The structure of SYSU-6 collapses and transforms into dense tridymite upon calcination at 550 °C (SYSU-6-cal). The preferred orientation effect is responsible for the differences in intensity. Scanning electron microscopy (SEM) imagery in Figure 1b shows that SYSU-6 had a long rod-like morphology, without obvious impurity observed. Element mappings (Figure 1c) indicate that Al, P, O were distributed among the crystals homogeneously. As presented in Figure 1d,e, transmission electron microscopy (TEM) images show the layers of structures in the framework. However, it is hard to identify the lattice fringes of the layers, which were possibly destroyed under the electron beams.

The infrared (IR) spectrum of SYSU-6 is shown in Figure S2. Several weak bands due to the C-H stretching modes of the ada molecules appeared in the range of 1250–1650 cm^−1^ and 2600–3000 cm^−1^. The bands at 1126, 1061, 466 cm^−1^ are associated with the phosphate oxoanion, and represent P–O or Al–O bending modes [19]. The other typical peaks appearing at 637 cm^−1^ arose from asymmetric stretching vibration of the P–O–Al unit, suggesting that AlPOs material was formed. Accordingly, the UV Raman spectrum (Figure S2) shows a typical aluminophosphate, with bands below 700 cm^−1^ corresponding to the Al–O–P structural species and a set of peaks in the range of 900–1100 cm^−1^ originating with the stretching modes of PO_4_ [20,21].

Single-crystal XRD analysis indicated that SYSU-6 crystallized in the space group *P*2_1_/*n*, with detailed information as seen in Table 2. Table 3 shows the final atomic coordinates and isotropic temperature factors. Selected bond lengths and bond angles are listed in Table 4.

**Figure 2:**
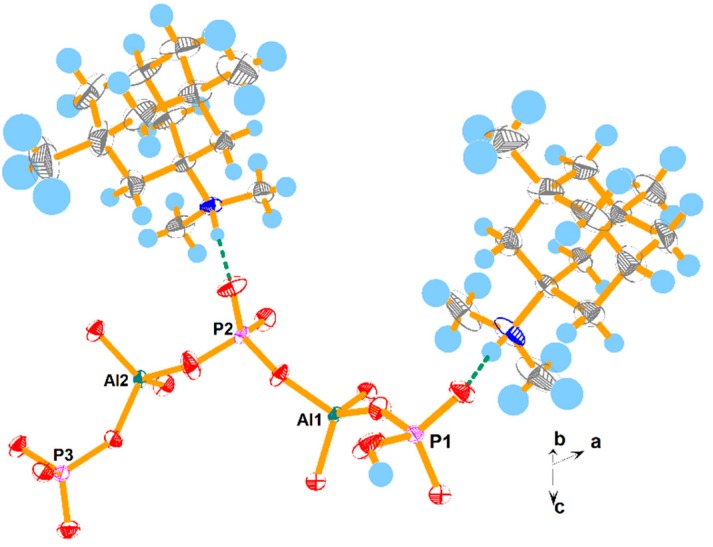
The asymmetric unit of SYSU-6, thermal ellipsoids at 50% probability. The hydrogen bonds are displayed as green dashed lines.

The structure of SYSU-6 contains macroanionic sheets [Al_2_(HPO_4_)(PO_4_)_2_]^2−^ that are charge-balanced by the protonated *N*,*N*,3,5-tetramethyladamantan-1-amine. The asymmetric unit (Figure 2) contained two tetrahedral Al sites, three tetrahedral P, and two OSDA molecules. As seen in Table 4, the Al–O bond lengths were between 1.714–1.740 Å and the O–Al–O angles were between 106.5–112.4°, which are typical for aluminophosphate materials. Of the three crystallographically distinct P sites, P–O bonds were in the range of 1.480–1.544 Å and the O–P–O were in the range of 105.6–112.1°. P1 had a hydroxyl group and a P=O double bond, characterized by the longer P1–O3 (1.544 Å) and shorter P1–O1 (1.480 Å) bond distance respectively. P2 and P3 share three oxygen with Al, leaving one oxygen terminal, that is, P2–O6 (1.481 Å) and P3–O6 (1.494 Å) respectively. The shorter length implies the double bond nature of both bonds.

**Figure 3:**
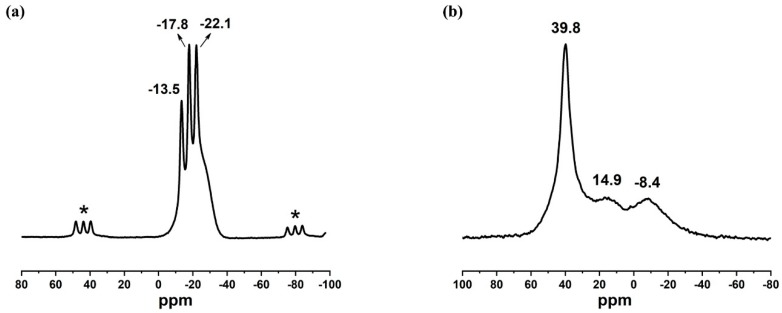
(**a**) ^27^Al magic angle spinning (MAS) NMR spectra of SYSU-6; (**b**) ^31^P MAS NMR spectra of SYSU-6. Asterisks (*) denote the spinning sidebands.

Local structures of aluminum and phosphorus in SYSU-6 were studied using ^27^Al and ^31^P magic angle spinning (MAS) NMR. As shown in Figure 3a, there were three peaks at −13.5 ppm, −17.8 ppm and −22.1 ppm. Previous studies [22,23] have shown that there are peaks at −13.5 ppm to P1, −17.8 ppm to P2, and −22.1 ppm to P3. This result was in accord with the presence of three inequivalent monophosphate sites in the structure of SYSU-6. The aluminum present in the SYSU-6 framework is mostly tetrahedrally coordinated, as a sharp resonance at 39.8 ppm was observed from the ^27^Al MAS NMR spectrum (Figure 3b). It should be noted that there were two weak signals at ca. 14.9 ppm and −8.4 ppm, suggesting that in the sample there was a small amount of five-coordinate and six-coordinate aluminum, respectively, which was attributed to extra-framework Al species.

**Figure 4:**
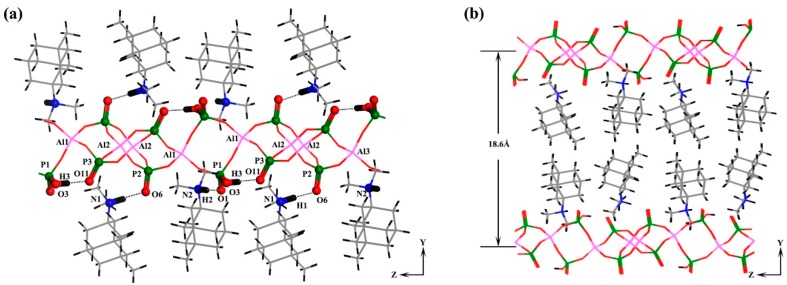
(**a**) A view of the intra-layer and inter-layer hydrogen-bond interactions; (**b**) Two layers of organic structure-directing agent (OSDA) molecules separate the aluminophosphate layers.

As seen in Figure 4a, H-bonding was found both intra-layer and inter-layer. The hydroxyl group P1–O3–H3 and P3–O11, where the distance of O3–O11 was 2.50 Å and the angle of O3–H3···O11 was 158.9°. Strong H-bonding was also found between the inorganic layer and OSDA molecules. The terminal P3=O11 and P2=O6 double bonds provide the H-bonding accepters, whereas, the protonated nitrogen atoms N1 and N2 of OSDA amine provide H-bonding donors to give N1–H1···O6 and N2–H2···O1 with H-bond lengths of 2.641 Å and 2.658 Å and H-bond angles of 176.7° and 175.9° respectively.

As seen in Figure 4b, unlike other aluminophosphate layers, the two inorganic layers A and B were completely separated by two layers of OSDA molecules with a distance of 18.6 Å to give a large void between the inorganic layers.

**Figure 5:**
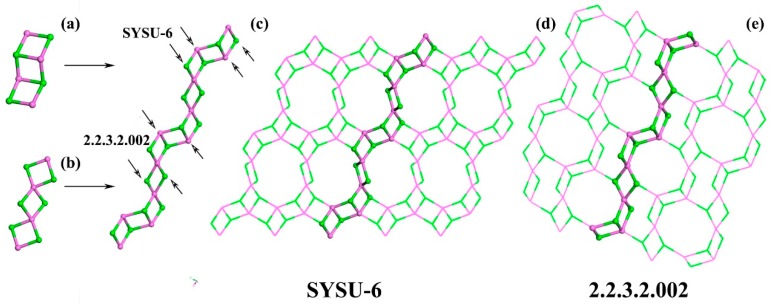
(**a**) Characteristic building unit “3 step ladder”; (**b**) Characteristic building unit “diamond telescopic hanger”; (**c**) Different connection way of the 4-ring infinite chain; (**d**) The layer of SYSU-6. (**e**) The layer of 2.2.3.2.002. Oxygen is omitted due to clarity.

Generally speaking, the inorganic layer of SYSU-6 can be constructed by a 4-ring infinite chain. A “3-step ladder” (Figure 5a) and “diamond telescopic hanger” (Figure 5b) are found to be the building units of the chain. The “diamond telescopic hangers” (Al_4_P_6_) link to each other to form an Al_2_P_3_ infinite chain. (Figure 5c). Interesting, this chain is found in the layers of both SYSU-6 (Figure 5d) and 2.2.3.2.002 [16] (Figure 5e). The only difference is the way to link them together. SYSU-6 is constructed by linking the neighboring Al–P pairs in the “3 step ladder”. Whereas, 2.2.3.2.002 is constructed by alternatively bonding one Al in the "3-step ladder" of one chain with one P of the “telescopic diamond hanger” of the adjacent chain. Therefore, the 12-ring pore and 8-ring pore are formed in SYSU-6 and 2.2.3.2.002, respectively.

## 3. Materials and Methods

We performed synthesis of *N*,*N*,3,5-tetramethyladamantan-1-amine. 21.8 g 3,5-dimethyl-1-adamantanamine hydrochloride (99%, HWRK Chem, Beijing, China) was fist deprotonated by NaOH aqueous solution, then the upper layer was extracted three times with ethyl acetate. After solvent removal by rotavapor, pale yellow liquid of 3,5-Dimethyl-1-adamantane (16.1 g, yield: 90.1%) was obtained. Next, the mixture of 3,5-dimethyl-1-adamantane (16.1 g, 0.09 mol), formic acid (88%, 14.1 g, 0.27 mol) and formaldehyde solution (37%, 21.9 g, 0.27 mol) were added to a 250 mL flask and stirring and heating at reflux temperature 98 °C for 18 h. After cooling, the mixture was alkalized with NaOH aqueous solution (30%) to range 12–13, and the organic phase was extracted with methylene dichloride three times. Then the separated phases were washed with a saturated sodium chloride solution twice and dried with anhydrous Na_2_SO_4_ and Na_2_CO_3_. After the solvent was distilled off, *N*,*N*,3,5-tetramethyladamantane-1-amine (C_14_H_25_N, denoted ada) was obtained as a mixture of pale yellow oil and white crystals (22.7 g, yield: 91.2%). ^1^H NMR (400 MHz, DCl_3_): 2.26(s, 6H), 2.13 (m, 1H), 1.51 (s, 2H), 1.29(m, 8H), 1.09(m, 2H), 0.83(s, 6H).

We performed synthesis of SYSU-6. Ada was used as the organic structure-directing agent, orthophosphoric acid (85 wt% H_3_PO_4_, Aladdin, Shanghai, China) and boehmite (Catapal B, 70.3% Al_2_O_3_, letai, Tianjin, China) were used as sources of phosphorus and aluminum, respectively. In a typical synthesis, 145 mg of boehmite was mixed with 1.50 g of distilled water and 354.9 mg of orthophosphoric acid and stirred for 2 h. Then, 419 mg of ada was introduced into the mixture and stirred for another 30 min to ensure homogeneity. The resulting gel, with a molar composition of Al_2_O_3_:1.5 P_2_O_5_:0.5 ada:20 H_2_O, was sealed in a 23 mL Teflon-lined stainless-steel autoclave, and then heated at 150 °C for 3 days under static conditions. The rodlike crystalline obtained was filtered and washed with water and ethanol, then dried at 80 °C overnight.

Elemental analysis shows a sample with an experimental C/N ratio of 14.7, which is close to the theoretical value 14.0 of ada. The ignorable discrepancy between the solid-state ^13^C MAS NMR of SYSU-6 with the liquid ^13^C NMR of the protonated ada (Figure S3) suggests that the template remained mostly intact in the pores.

For characterization, single-crystal diffraction data were recorded on a D8 QUEST diffractometer (Bruker, Karlsruhe, Germany) with Mo*K_α_* (λ = 0.71073 Å) radiation in sequence at 300 K for |Hada|_2_[Al_2_(HPO_4_)(PO_4_)_2_]. The crystal structures were solved by direct methods, and all non-hydrogen atoms were refined anisotropically by least-squares on *F*^2^ using the SHELXTL 2014/7 program [24]. Hydrogen atoms on organic ligands were generated by the riding mode. The responses to the alerts from checkCIF are quoted within the validation response form. The Cambridge Crystallographic Data Centre (CCDC) 1937152 contains the supplementary crystallographic data for this paper. These data could be obtained free of charge from the Cambridge Crystallographic Data Centre via www.ccdc.cam.ac.uk/data_request/cif.

PXRD was conducted using a SmartLab diffractometer (Rigaku Corporation, Tokyo, Japan) equipped with a rotating anode (Cu *Kα*_1_ radiation, λ = 1.5406 Å). SEM images and corresponding energy-dispersive X-ray analysis (EDX) were obtained using a Quanta 400 Thermal Field Emission Environmental SEM (FEI Company, Hillsboro, OR, USA). TEM images were acquired on a JEM-ARM200P (JEOL, Tokyo, Japan), operating at 200 kV. The UV Raman spectroscopy was recorded in the range of 200–1200 cm^−1^ nm on LabRAM HR Evolution (Horiba, Paris, France). The IR spectrum was collected within the range of 4000–400 cm^−1^ on Nicolet 6700 (Thermo Scientific, Waltham, MA, USA) Fourier transform infrared (FI-IR) spectroscopy. Elemental analysis was performed on a Vario-ELCHNS elemental analyzer (Elementar Analyzensysteme GmbH, Hanau, Germany). The solid-state ^27^Al, ^31^P, and ^13^C MAS NMR spectra were collected using a Bruker Advance 400 spectrometer with 79.49 MHz (Bruker BioSpin, Switzerland). The liquid ^13^C-NMR spectrum was recorded on a Bruker advance III 400MHz spectrometer (Bruker BioSpin, Fällanden, Switzerland).

## 4. Conclusions

This work has presented and characterized a 2-D aluminophosphate·|Hada|_2_[Al_2_(HPO_4_)(PO_4_)_2_] with new topology. The layer characterized by a 4, 12 ring net interwoven with a chain composed of an edge-sharing 4-ring and vertex-sharing 4-ring. Furthermore, the structural similarity and difference, that is, the different ways to connect a common chain, was discussed. This new structure has the potential to further set up a 3-D framework zeolite through condensation of additional Al unit bridges between the two layers.

## Figures and Tables

**Table 1:** Summary of Layered aluminophosphates (AlPO) with Al:P Ratio of 2:3.

Structure Code ^1^	Amine	*x* ^3^	Polyhedra Building Units	Stacking Sequence	Ring Size	Ref
2.2.3.14.001	cyclohexylamine	1	AlO_4b_, PO_2b_O_2t_, PO_3b_O_1t_	AA	4,6,8	[9]
2.2.3.14.002	cyclohexylamine	1	AlO_4b_, PO_2b_O_2t_, PO_3b_O_1t_	AB	4,6	[9]
2.2.3.14.003	cyclopentylamine	1	AlO_4b_, PO_2b_O_2t_, PO_3b_O_1t_	AB	4,6	[10]
2.2.3.14.004	2-butylamine	1	AlO_4b_, PO_2b_O_2t_, PO_3b_O_1t_	AB	4,6	[11]
2.2.3.14.005	4-methylpyridine	2	AlO_4b_, AlO_5b_, PO_2b_O_2t_, PO_3b_O_t_, PO_4b_	AB	4,6	[12]
2.2.3.14.006	Cs^+^ ions	2	AlO_6b_, PO_3b_O_t_, PO_4b_	AA	4,8	[13]
2.2.3.14.007	*tert*-butylamine	1	AlO_4b_, PO_2b_O_2t_, PO_3b_O_1t_	AB	4,6	[14]
2.2.3.15.001	tri-*n*-propylamine	2	AlO_4b_, PO_2b_O_2t_, PO_3b_O_1t_	ABCD	4,8	[15]
2.2.3.2.001	pyridine	2	AlO_4b_, AlO_5b_, PO_2b_O_2t_, PO_3b_O_1t_, PO_4b_	AA	4,6,8	[11]
2.2.3.2.002	2,2,6,6-tetramethylpiperidine	2	AlO_4b_, PO_2b_O_2t_, PO_3b_O_1t_	AA	4,8	[16]
2.2.3.2.003	Co-Tet-A ^2^	1	AlO_4b_, PO_3b_O_1t_, CoO_2b_N_4_	AA	4	[17]
2.2.3.33.001	*N*-methyl-ethylenediamine	2	AlO_4b_, AlO_5b_O_1t_, PO_3b_O_1t_	AB	4,8	[18]
2.2.3.33.002	*N*-methyl-1,3-propanediamine	2	AlO_4b_, AlO_5b_O_1t_, PO_3b_O_1t_	AB	4,8	[18]
SYSU-6	*N*,*N*,3,5-tetramethyladamantan-1-amine	2	AlO_4b_, PO_2b_O_2t_, PO_3b_O_1t_	AB	4,12	This work

^1^ Structure code is taken from a database from Li and Yu [7]. ^2^ Al_4_(PO_4_)_4_(HPO_4_)_2_[Co(C_16_H_36_N_4_)]C_16_H_38_N_4_·2H_2_O, where C_16_H_36_N_4_ is meso-5,7,7,12,14,14-hexamethyl-1,4,8,11-tetraazacyclotetradecane. ^3^ *x* Value in formula |OSDA^n+^|_(3–*x*)/*n*_ [Al_2_(HPO_4_)*_x_*(PO_4_)_3–*x*_].

**Table 2:** Crystal data and structure refinement for SYSU-6.

Empirical Formula	C_28_H_53_N_2_Al_2_P_3_O_12_	ρ_calc_g/cm^3^	1.344
Formula weight	756.59	μ/mm^−1^	0.264
Temperature/K	300.01	F(000)	1608.0
Crystal system	monoclinic	Crystal size/mm^3^	0.16 × 0.134 × 0.063
Space group	*P*2_1_/n	Radiation MoK_α_	λ = 0.71073
a/Å	8.4455(2)	Index ranges	−10 ≤ h ≤ 10, −48 ≤ k ≤ 44, −16 ≤ l ≤ 16
b/Å	37.1474(12)	2θ range for data collection/°	4.744 to 55.036
c/Å	12.5969(4)	Reflections collected	28080
α/°	90	Independent reflections	8515 [*R*_int_ = 0.0465, *R*_sigma_ = 0.0531]
β/°	108.8760(10)	Data/restraints/parameters	8515/6/433
γ/°	90	Goodness-of-fit on F^2^	1.023
Volume/Å^3^	3739.47(19)	Final *R* indexes [I > 2σ (*I*)]	*R*_1_ = 0.0512, *wR*_2_ = 0.1050
Z	4	Final R indexes [all data]	*R*_1_ = 0.0832, *wR*_2_ = 0.1194

**Table 3:** Fractional atomic coordinates (×10^4^) of Al, P, O, C, N and selected H and equivalent isotropic displacement parameters (Å^2^ × 10^3^) for SYSU-6. R_eq_ is defined as 1/3 of the trace of the orthogonalized U_IJ_ tensor.

Atom	x	y	z	Atom	x	y	z
P1	5923.4(8)	5557.3(2)	10,444.7(5)	C7	12,222(4)	6534.3(9)	8894(3)
P2	975.8(8)	5463.5(2)	6248.3(5)	C8	11,339(4)	6168.9(8)	8742(3)
P3	−4662.9(8)	4707.6(2)	6631.8(5)	C9	10,926(5)	6826.2(10)	8396(3)
Al1	3915.8(9)	5146.0(2)	8219.3(6)	C10	9563(5)	6838.8(9)	8959(3)
Al2	−2349.6(9)	5014.7(2)	5357.8(6)	C11	10,397(5)	6902.2(10)	10,214(3)
O1	7201(2)	5836.4(5)	10,508.1(15)	C12	13,565(5)	6525.8(12)	8322(4)
O2	6686(2)	5228.4(5)	11,174.4(15)	C13	8264(7)	7128.4(12)	8431(5)
O3	4466(3)	5698.4(7)	10,818.1(17)	C14	8723(4)	6466.9(9)	8803(3)
O4	5122(2)	5434.3(5)	9227.7(15)	C15	1593(4)	6062.3(9)	3778(3)
O5	2152(2)	5376.0(5)	7429.5(14)	C16	−1137(4)	5780.5(8)	3285(3)
O6	669(3)	5855.3(5)	6084.8(15)	C17	−877(4)	6436.0(7)	3870(2)
O7	1732(2)	5315.8(5)	5384.4(15)	C18	−2386(4)	6408.4(9)	4263(3)
O8	−642(2)	5259.4(6)	6125.2(17)	C19	−3217(5)	6782.1(11)	4210(4)
O9	−3114(2)	4802.4(5)	6315.8(15)	C20	−3690(5)	6915.9(12)	2979(4)
O10	−4902(2)	5014.7(5)	7390.3(14)	C21	−2171(6)	6947.0(11)	2616(3)
O11	−4474(2)	4349.8(5)	7200.4(15)	C22	−1396(5)	6572.5(9)	2655(3)
O12	−6179(2)	4707.2(5)	5547.0(15)	C23	−934(6)	7206.0(10)	3371(3)
N1	−48(3)	6066.3(6)	3979.5(18)	C24	−420(5)	7074.7(9)	4584(3)
N2	9072(4)	5813.4(7)	9171(3)	C25	363(4)	6699.6(8)	4626(3)
C1	10,153(6)	5495.9(10)	9572(6)	C26	−1951(5)	7041.9(10)	4949(3)
C2	7926(6)	5741.5(12)	7985(4)	C28	855(6)	7333.3(11)	5356(4)
C3	9998(4)	6172.7(7)	9303(2)	H1	181.79	5992.81	4762.17
C4	10,791(4)	6246.2(9)	10,559(3)	H2	8347.26	5829.33	9639.77
C5	11,659(5)	6611.3(10)	10,718(3)	H3	4722.3	5684.15	11,502.54
C6	13,005(5)	6610.4(11)	10,150(3)				

**Table 4:** Selected bond lengths and angles for SYSU-6.

Bond	Length/Å	Atom	Angle/˚	Atom	Angle/˚
P1–O1	1.4801(19)	O1–P1–O2	111.54(11)	O7 ^4^–Al2–O9	107.48(10)
P1–O2	1.5393(18)	O1–P1–O4	109.94(11)	O8–Al2–O12 ^3^	110.40(11)
P1–O4	1.5314(18)	O1–P1–O3	112.62(14)	O8–Al2–O9	106.48(10)
P1–O3	1.544(2)	O2–P1–O3	107.95(11)	O8–Al2–O7 ^4^	109.67(11)
P2–O5	1.5329(17)	O4–P1–O2	108.97(11)	P3–O10–Al1 ^5^	142.93(12)
P2–O7	1.5305(18)	O4–P1–O3	105.58(12)	P2–O5–Al1	146.26(12)
P2–O8	1.526(2)	O7–P2–O5	109.02(10)	P3–O12–Al2 ^3^	142.84(13)
P2–O6	1.4810(19)	O8–P2–O5	105.72(11)	P1–O2–Al12	140.49(12)
P3–O10	1.5425(18)	O8–P2–O7	108.38(12)	P3–O9–Al2	146.18(13)
P3–O11	1.4939(19)	O6–P2–O5	111.87(11)	P1–O4–Al1	152.72(14)
P3–O12	1.5411(18)	O6–P2–O7	110.34(11)	P2–O7–Al2 ^4^	155.56(14)
P3–O9	1.5264(19)	O6–P2–O8	111.33(13)	P2–O8–Al2	152.00(14)
Al1–O10 ^1^	1.7322(18)	O11–P3–O10	112.09(10)	C16–N1–C17	114.2(2)
Al1–O5	1.7264(18)	O11–P3–O12	110.68(11)	C15–N1–C17	114.8(2)
Al1–O2 ^2^	1.7397(18)	O11–P3–O9	111.28(11)	C15–N1–C16	109.0(3)
Al1–O4	1.7212(19)	O12–P3–O10	108.21(10)	C2–N2–C3	113.6(3)
Al2–O12 ^3^	1.7304(19)	O9–P3–O10	106.60(11)	C1–N2–C3	115.0(3)
Al2–O9	1.7319(19)	O9–P3–O12	107.78(10)	C1–N2–C2	108.8(4)
Al2–O7 ^4^	1.7232(19)	O10 ^1^–Al1–O2 ^2^	110.31(9)	N2–C3–C4	107.8(2)
Al2–O8	1.714(2)	O5–Al1–O10 ^1^	110.50(9)	C8–C3–N2	112.0(2)
N1–C17	1.528(3)	O5–Al1–O2 ^2^	109.18(9)	C8–C3–C14	110.5(2)
N1–C16	1.490(4)	O4–Al1–O10 ^1^	107.51(10)	C8–C3–C4	109.9(2)
N1–C15	1.487(4)	O4–Al1–O5	108.16(10)	C14–C3–N2	108.3(2)
N2–C3	1.528(4)	O4–Al1–O2 ^2^	111.15(9)	N1–C17–C22	111.2(2)
N2–C2	1.519(5)	O12 ^3^–Al2–O9	112.38(10)	C25–C17–N1	108.6(2)
N2–C1	1.476(5)	O7 ^4^–Al2–O12 ^3^	110.31(9)	C25–C17–C22	109.1(3)

Symmetry transformations used to generate equivalent atoms are as follows: ^1^ 1 + X,+Y,+Z; ^2^ 1 − X,1 − Y,2 − Z; ^3^ −1 − X,1 − Y,1 − Z; ^4^ −X,1 − Y,1 − Z; ^5^ −1 + X,+Y,+Z.

## Supplementary Materials

The Supplementary Materials are available online. Figure S1: IR spectrum of SYSU-6 with the range of 400–4000 cm^−1^; Figure S2: UV Raman spectrum excited with 325 nm of SUSY-6 with the range of 200–1200 cm^−1^; Figure S3: The solid-state ^13^C MAS NMR of SYSU-6 and the ^13^C Liquid NMR of synthetic protonated *N*,*N*,3,5-tetramethyladamantan-1-amine dissolved with concentrated HCl aqueous solution.
