# Supplementary material for: SYSU-6, A New 2-D Aluminophosphate Zeolite Layer Precursor
Source: Molecules. 2019 Aug 16;24(16):2972. doi: 10.3390/molecules24162972 (PMC6720306; doi:10.3390/molecules24162972)
Supplement: Supplementary file 1 [file molecules-24-02972-s001.zip › molecules-551069-SI.pdf]

Supporting Information

SYSU-6, A new 2-D Aluminophosphate Zeolite Layer Precursor

Jiang-Zhen Qiu, Long-Fei Wang, Jiuxing Jiang*

MOE Key Laboratory of Bioinorganic and Synthetic Chemistry, School of Chemistry, Sun Yat-sen University, Guangzhou, 510275, P. R. China. E-mail: jiangjiux@mail.sysu.edu.cn

Correspondence: jiangjiux@mail.sysu.edu.cn; Tel.: +86-20-84111355

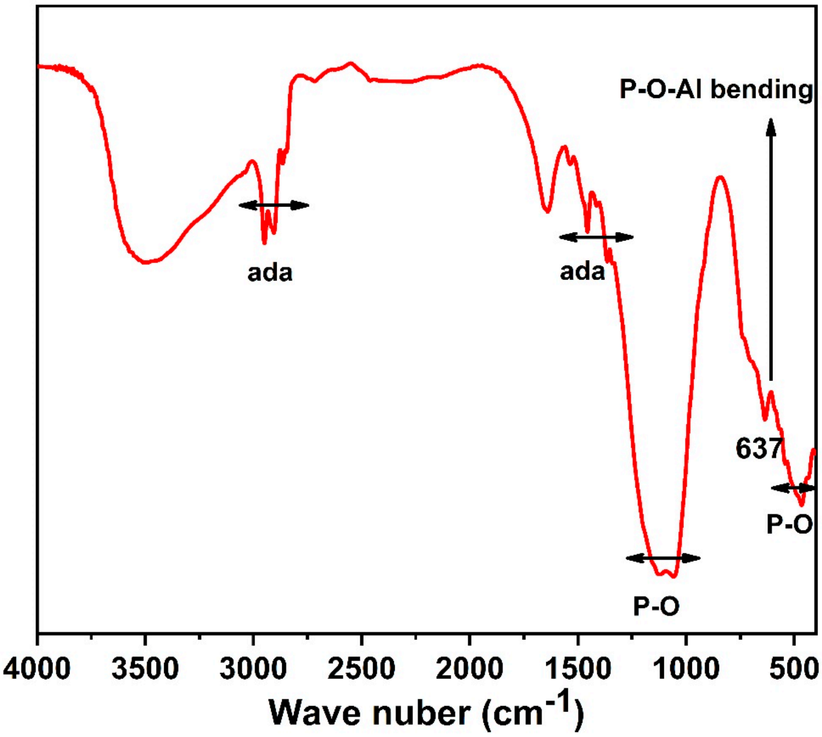

Figure S1. IR spectrum of SYSU-6 with the range of 400-400 cm^{-1}.

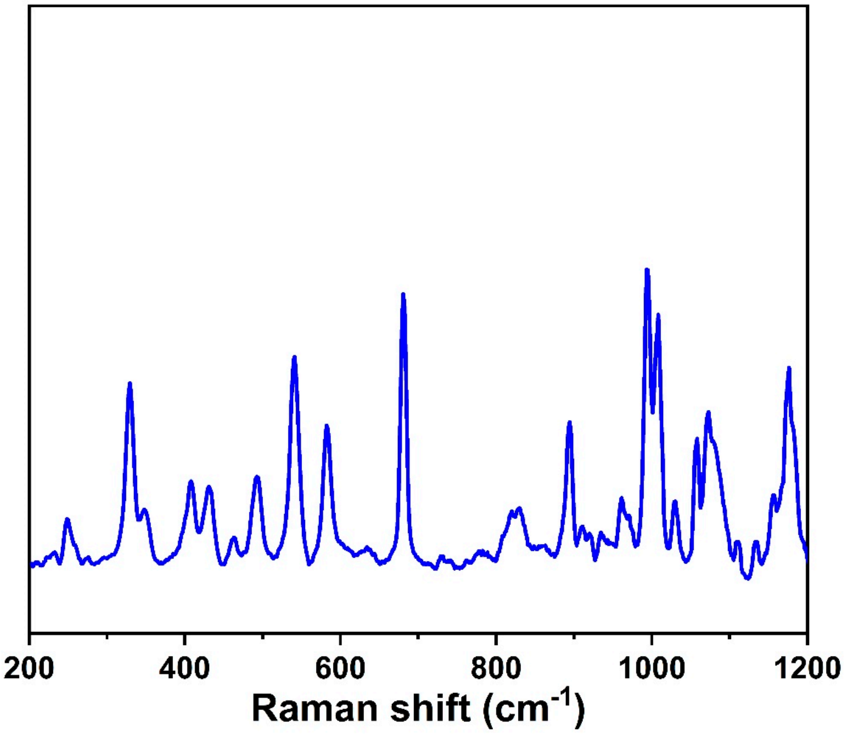

Figure S2. UV Raman spectrum excited with 325 nm of SUSY-6 with the range of 200–1200 cm^{-1}.

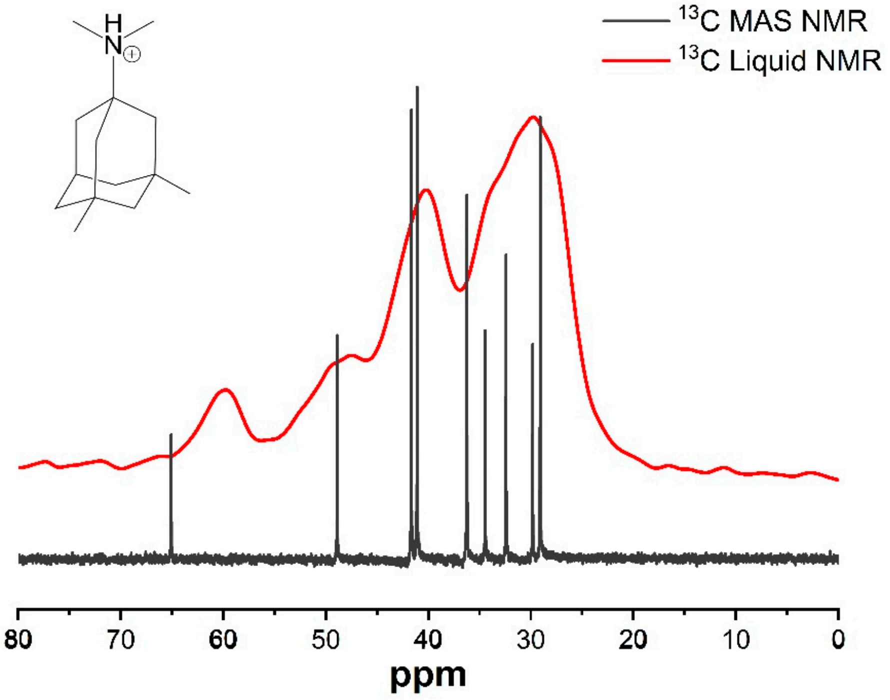

Figure S3. The solid-state ^{13}C MAS NMR of SYSU-6 and the ^{13}C Liquid NMR of synthetic protonated N,N,3,5-tetramethyladamantan-1-amine dissolved with concentrated HCl aqueous solution.
